# Deciphering Additional Roles for the EF-Tu, l-Asparaginase II and OmpT Proteins of Shiga Toxin-Producing *Escherichia coli*

**DOI:** 10.3390/microorganisms8081184

**Published:** 2020-08-04

**Authors:** Alexia N. Torres, Nayaret Chamorro-Veloso, Priscila Costa, Leandro Cádiz, Felipe Del Canto, Sebastián A. Venegas, Mercedes López Nitsche, Roberto F. Coloma-Rivero, David A. Montero, Roberto M. Vidal

**Affiliations:** 1 Programa de Microbiología y Micología, Instituto de Ciencias Biomédicas, Facultad de Medicina, Universidad de Chile, Santiago 8380453, Chile; ale.ntm6@gmail.com (A.N.T.); nayarethalseide@gmail.com (N.C.-V.); priscilasc88@hotmail.com (P.C.); lecadiz13@gmail.com (L.C.); felipedelcanto@uchile.cl (F.D.C.); sebastian.venegas.g4@gmail.com (S.A.V.); 2 Programa Disciplinario de Inmunología, Instituto de Ciencias Biomédicas, Facultad de Medicina, Universidad De Chile, Santiago 8380453, Chile; melopez@uchile.cl; 3 Instituto Milenio de Inmunología e Inmunoterapia, Facultad de Medicina, Universidad de Chile, Santiago 8380453, Chile; 4 Departamento de Microbiología, Facultad de Ciencias Biológicas, Universidad de Concepción, Concepción 4030000, Chile; rcoloma@udec.cl; 5 Núcleo Milenio Biología de Microbiota Intestinal, Santiago 8320000, Chile

**Keywords:** EF-Tu, l-asparaginase II, OmpT, Shiga toxin-producing *Escherichia coli*, STEC.

## Abstract

Shiga toxin-producing *Escherichia coli* (STEC) causes outbreaks and sporadic cases of gastroenteritis. STEC O157:H7 is the most clinically relevant serotype in the world. The major virulence determinants of STEC O157:H7 are the Shiga toxins and the locus of enterocyte effacement. However, several accessory virulence factors, mainly outer membrane proteins (OMPs) that interact with the host cells may contribute to the virulence of this pathogen. Previously, the elongation factor thermo unstable (EF-Tu), l-asparaginase II and OmpT proteins were identified as antigens in OMP extracts of STEC. The known subcellular location of EF-Tu and l-asparaginase II are the cytoplasm and periplasm, respectively. Therefore, we investigate whether these two proteins may localize on the surface of STEC and, if so, what roles they have at this site. On the other hand, the OmpT protein, a well characterized protease, has been described as participating in the adhesion of extraintestinal pathogenic *E. coli* strains. Thus, we investigate whether OmpT has this role in STEC. Our results show that the EF-Tu and l-asparaginase II are secreted by O157:H7 and may also localize on the surface of this bacterium. EF-Tu was identified in outer membrane vesicles (OMVs), suggesting it as a possible export mechanism for this protein. Notably, we found that l-asparaginase II secreted by O157:H7 inhibits T-lymphocyte proliferation, but the role of EF-Tu at the surface of this bacterium remains to be elucidated. In the case of OmpT, we show its participation in the adhesion of O157:H7 to human epithelial cells. Thus, this study extends the knowledge of the pathogenic mechanisms of STEC.

## 1. Introduction

Moonlighting proteins are a group of proteins that have multiple and non-mutually exclusive functions, which can be performed in different locations within or outside the cell. The multitasking capabilities of these proteins are not due to gene fusions, splice variants or post-translational modifications. Rather, the variation in their functions is attributed to changes in the subcellular localization, oligomerization, use of distinct binding sites or variations in the cellular concentration of a ligand, cofactor or product [1,2]. In recent years, it has been shown that some bacterial moonlighting proteins involved in metabolism or stress response also have a virulence-related function, participating in adhesion, invasion, modulation and evasion of host immune responses [3,4,5,6]. Consequently, there has been an increasing interest in studying this class of proteins in many bacterial pathogens.

Shiga toxin-producing *Escherichia coli* (STEC) are a group of zoonotic and foodborne pathogens that cause gastrointestinal infections with a range of clinical outcomes, from acute diarrhea to bloody diarrhea and life-threatening diseases such as hemolytic uremic syndrome (HUS) [7]. Analyses of the STEC proteome have revealed a number of moonlighting proteins which could contribute to the pathogenesis of this bacterium. For instance, NAD-dependent glyceraldehyde-3-phosphate dehydrogenase (GAPDH), a cytoplasmic protein involved in the glycolysis pathway, is secreted by STEC O157:H7 to the culture medium, where it may act as an adhesin that binds to fibrinogen and epithelial cells [8]. Enolase is another moonlighting protein with a possible role in the pathogenesis of STEC [9].

In a previous study, we performed an immunoproteomic analysis of outer membrane protein (OMP) extracts of several STEC serotypes and identified a number of proteins that are reactive to sera from patients who developed HUS [10]. Most of the identified antigens were integral membrane proteins such as OmpA, OmpC, OmpF and OmpT. However, the elongation factor Tu (EF-Tu) and l-asparaginase II were also antigens identified in the OMP extracts. Since these two proteins lack a recognizable signal sequence for surface anchoring, secretion or translocation to the outer membrane, their identification was unexpected.

EF-Tu, encoded by the *tuf* gene, is a protein involved in several functions in the cytoplasm, including the transport of aminoacylated tRNAs to the ribosome, protein folding, protection from stress and functioning as an actin-like cytoskeletal element [11,12,13,14]. In addition, it has been demonstrated that this protein may also localize on the surface of some bacteria, where it may play a role in colonization-associated phenotypes or even in virulence [4,5]. Depending on the microorganism, EF-Tu may act as an adhesin with binding capabilities to host proteins, including fibrinogen [15,16,17], fibronectin [17,18,19,20,21,22,23], laminin [23], plasminogen [15,16,24], fetuin [25], factor H [15,16,24,26] and mucin [27,28,29]. A recent study showed the localization of EF-Tu on the bacterial surface of some uropathogenic *E. coli* (UPEC) strains and suggested that this protein could promote kidney stone formation [30]. In the case of diarrheagenic *E. coli* (DEC), the localization of EF-Tu on the bacterial surface and a possible role in virulence have not yet been demonstrated.

On the other hand, l-asparaginase is an enzyme that catalyzes the hydrolysis of L-asparagine to provide the bacterium with a nutritional source of nitrogen, aspartic acid and ammonia [31,32]. *E. coli* contains two l-asparaginase isoenzymes encoded by two different genes: the l-asparaginase I localized in the cytoplasm encoded by the *ansA* gene, and the l-asparaginase II localized in the periplasm encoded by the *ansB* gene. Both l-asparaginases are involved in the metabolism of l-asparagine, but type II has a greater affinity with the substrate [32]. Interestingly, recent studies have shown that l-asparaginase can function beyond its metabolic role by promoting the infectivity of some pathogens. For instance, l-asparaginase II secreted by *Salmonella* Typhimurium reduces the amount of exogenous L-asparagine, causing T cell blastogenesis suppression and T cell response inhibition [31,33,34]. In *E. coli*, it is unknown whether this enzyme has a role in virulence.

As mentioned, we also identified the OmpT protein as an antigen of STEC. The product of the *ompT* gene is a protease with a well-known role in the virulence of extraintestinal pathogenic *E. coli* (ExPEC), avian pathogenic *E. coli* (APEC) as well as in DEC strains [35,36,37,38,39,40,41].

The proteolytic activity of OmpT mediates resistance against antimicrobial peptides such as protamine and cathelicidin LL-37 [35,36,37], and it is involved in the biogenesis of outer membrane vesicles (OMVs) [38]. In addition, some studies have suggested that OmpT participates in the adhesion of ExPEC and APEC strains [39,40,41]; however, whether this additional role depends on its proteolytic activity has not been demonstrated conclusively to date.

In the current study, we investigate possible novel functions and biological roles of the EF-Tu, l-asparaginase II and OmpT proteins that could contribute to the virulence of STEC. As increasingly shown for other pathogens, we hypothesize that in STEC, EF-Tu and l-asparaginase II are secreted and located on the bacterial surface, where they may act as virulence factors. In the case of OmpT, we focus our efforts on determining whether this protein participates in the adhesion of STEC. Our results contribute to the knowledge of STEC pathogenic mechanisms and to deciphering the non-canonical roles of these proteins.

## 2. Materials and Methods

### 2.1. Bacterial Strains

STEC O157:H7 str. EDL933 (ATCC 43895) is a food isolate from a 1982 hemorrhagic colitis outbreak in Michigan [42]. *E. coli* DH5α was used for the generation and manipulation of plasmids. Strains were routinely grown in Luria-Bertani (LB) broth or Dulbecco’s modified Eagle medium (DMEM) at 37 °C with agitation. Bacteriological agar in a final concentration of 1.5% (wt/vol) was added to prepare the solid media. The culture media were supplemented as needed with ampicillin (100 µg/mL), kanamycin (50 µg/mL), and 2 mM m-toluic acid. For the proliferation assay, the strain *Salmonella* Typhimurium (ATCC 14028) was used as the positive control (kindly provided by Dr. Carlos Santiviago, University of Chile). Another two strains were used as the control for a comparative proteomic analysis of the OMVs: the STEC O91:H21 str. V07-4-4 [43] and the AIEC O83:H1 str. NRG857c [44], kindly provided by Dr. Alfredo Torres from the University of Texas Medical Branch (UTMB).

### 2.2. Construction of Isogenic Mutants

The *ansB* and *ompT* genes were inactivated in the EDL933 strain (GenBank accession No. AE005174.2) by using the lambda red recombinase system as described by Datsenko and Wanner [45]. Briefly, the EDL933 strain was transformed using the plasmid pKD46. Then, a kanamycin-resistance gene was amplified from plasmid pKD4 using the primer pairs d-ompT_F + d-ompT _R and d-ansB _F + d-ansB _R (Table 1). The PCR products were purified and electroporated into the EDL933 strain carrying the plasmid pKD46. The kanamycin-resistant clones were analyzed by PCR to verify the allelic exchange using an internal primer to the *kan* gene (kanR_R) and another flanking the *ansB* (ansB-e _F) and *ompT* (ompT-e _F) genes. Finally, these PCR products were sequenced for further confirmation and plasmid pKD46 was cured from the isogenic mutants by overnight growth at 37 °C.

### 2.3. Cloning and Expression of the ansB and ompT Genes

The *ansB* and *ompT* genes were amplified from the EDL933 genome using the primer pairs ansBc_F + ansBc_R and ompTc_F + ompTc_R, respectively (Table 1). These primer pairs made it possible to obtain PCR products with recognition sites for the restriction enzymes NdeI and BamHI in the 5′ and 3′ ends of each gene, respectively. PCR products were ligated to the plasmid pTZ57R/T (Fermentas, Vilnius, Lithuania) to obtain the derived plasmids pTZ57R/T_*ansB* and pTZ57R/T_*ompT*. Then, these vectors were electroporated into the *E. coli* DH5α, and clones were selected according to Amp resistance and α-complementation. The correct clone was confirmed by sequencing (Macrogen, Rockville, MD, USA). Next, the corresponding plasmids were extracted from the transformed *E. coli* DH5α strains and digested with NdeI and BamHI. The digestion products were analyzed by agarose gel electrophoresis, and the inserts (*ansB* and *ompT* genes) were purified and ligated to the plasmid pVB1 (Dualsystems Biotech, Schlieren, Switzerland), in which the inserted gene was regulated by the Pm/xylS expression system. The pVB1 is a low-copy-number plasmid that enables the control and expression of a cloned gene by supplementing the media with 2mM m-toluic acid. The derived plasmids pVB1_*ansB* and pVB1_*ompT* were used to transform the EDL933∆*ansB* and EDL933∆*ompT* strains, respectively. The expression of the cloned genes was induced by supplementing the culture medium with 2 mM m-toluic acid.

### 2.4. Western Blotting

The bacteria were grown for 18–20 h in DMEM medium supplemented as required with the corresponding antibiotic and 2 mM m-toluic acid. The culture supernatant was collected, and secreted proteins were precipitated with trichloroacetic acid (TCA) by incubation overnight at 4 °C, followed by centrifugation at 15,000× *g* at 4 °C for 30 min. The pellets were suspended in 10 mM Tris-HCl buffer (pH 7.5). The outer membrane protein (OMPs) extracts were obtained as previously described [10]. Protein extracts were analyzed by SDS-PAGE and then transferred to nitrocellulose membranes using standard procedures. Membranes were blocked with blocking solution (1X Tris-buffered saline [TBS], 0.003% Tween 20, 1% BSA) for 1 h at room temperature. Following three washes with TBS-T (1X TBS-0.03% Tween 20) solution for 5 min each time, the membranes were incubated for 1 h at room temperature with agitation in a blocking solution containing the corresponding primary antibody. The EF-Tu, l-asparaginase II and OmpT proteins were detected using the mouse IgG monoclonal anti-EF-Tu antibody 900 (dilution 1:2000; Cat # HM6010, HyCult Biotechnology, Uden, The Netherlands), rabbit IgG anti-asparaginase antibody (dilution 1:2000; Cat # ab135225, Abcam, Cambridge, UK) and rabbit IgG anti-OmpT antibody (dilution 1:3000; Cat # orb51410, Biorbyt LLC, San Francisco, CA, USA), respectively. After two washes with TBS-T solution for 10 min each time, the membranes were incubated for 1 h at room temperature with agitation in a blocking solution containing goat anti-mouse IgG, HRP conjugated (dilution 1:5000, Cat # G-21040, ThermoFisher Scientific, San Jose, CA, USA) or goat anti-rabbit IgG, HRP conjugate (dilution 1:5000, Cat # G-21234, ThermoFisher Scientific, USA). Following two washes with TBS-T for 10 min each time, membranes were revealed with Novex™ HRP Chromogenic Substrate (ThermoFisher Scientific, USA), and the reaction was stopped with distilled water.

### 2.5. Transmission Electron Microscopy (TEM) and Immunogold Labeling

TEM visualization and immunogold labeling were performed as previously described [46]. For detection of EF-Tu and l-asparaginase II proteins, the mouse IgG monoclonal anti-EF-Tu antibody 900 (dilution 1:200; Cat # HM6010, Hycult Biotech Inc, USA) and a rabbit IgG anti-Asparaginase antibody (dilution 1:200; Cat # ab135225, Abcam, UK) were used as the primary antibody, respectively. As secondary antibodies, gold-conjugated goat anti-mouse IgG and IgM antibody (10 nm gold particles) at a dilution of 1:100 and gold-conjugated anti-rabbit IgG (20 nm gold particles) were used. Samples were analyzed with a Philips Tecnai 12 microscope at the Electron Microscopy Laboratory, Faculty of Biological Sciences, Pontificia Universidad Católica de Chile.

### 2.6. Proteomic Analysis of Outer Membrane Vesicles (OMVs)

OMVs were purified using the ExoBacteria™ OMV Isolation Kit (System Biosciences, Palo Alto, CA, USA) following the manufacturer’s protocols. The proteomic analysis was carried out by the proteomics company Bioproximity (Manassas, VA, USA). Briefly, the protocol accessed was label-free, quantitative, shotgun proteomic profiling of affinity purified samples (LFQ Profiling, in-Solution-Top 500). Protein identification and quantitation of up to the 500 most abundant protein groups in the sample were carried out via UPLC-MS/MS on Q-Exactive Orbitrap HF-X. Unbiased, data-dependent acquisition was performed, inclusive of sample preparation via digestion, sequence library searching, spectral library matching, match-between-runs assignment, relative quantitation and reporting.

### 2.7. Bacterial Adhesion Assays

Bacterial adhesion to HT-29 (ATCC^®^ HTB-38™) cells was evaluated as previously described [47] with modifications. Briefly, epithelial cells were cultured in DMEM supplemented with 10% bovine fetal serum and 1% penicillin-streptomycin at 37 °C in 5% CO_2_ atmosphere. Cells were seeded in 24-well plates and grown to confluence (approximately 4 × 10^5^ cells/well). Bacterial pre-inoculates were grown overnight in DMEM low-glucose supplemented with the appropriate antibiotic and 2 mM m-toluic acid at 37 °C with agitation. An aliquot of each pre-inoculum was diluted 50-fold in the same supplemented culture medium and incubated at 37 °C for 4 h with agitation. The epithelial cells were washed three times with PBS and infected with a multiplicity of infection (MOI) of 10 for 3 h at 37 °C in 5% CO_2_ atmosphere. Non-adherent (planktonic) bacteria were collected and cell washed five times with PBS. Cell-associated (adherent) bacteria were recovered by lysis with 0.1% Triton X-100. The number of planktonic and adherent bacteria were determined by serial dilution and counts of viable bacteria in LB agar. Results were expressed as the percentage of cell-associated bacteria relative to the total bacteria present after infection (planktonic bacteria plus adherent bacteria).

Bacterial adhesion to fibronectin and laminin was evaluated as previously described [48].

To evaluate the possible participation of EF-TU in adhesion, before the infection, the EDL933 strain was incubated for 30 min at 37 °C with agitation, with the mouse IgG monoclonal anti-EF-Tu antibody 900 (Cat # HM6010, Hycult Biotech Inc, USA) at dilutions of 1:10 and 1:100 or purified mouse IgG (Cat #PM901, Bio-rad, USA) at dilutions of 1:10 and 1:100.

### 2.8. Proliferation Assay

T cells isolated from peripheral blood mononuclear cells (PBMCs) of healthy donors were obtained from the Blood Bank Service of the University of Chile Clinical Hospital [49]. Five hundred thousand irradiated PBMCs (stimulator) prepared from buffycoat and 0.5 × 10^6^ T cell allogeneic (responder) buffycoat were co-cultured in triplicate in 96-well plates at 37 °C in complete RPMI (Gibco BRL, Grand Island NY) with 1% penicillin-streptomycin, 1% l-glutamine, and 5% human AB serum (Gemini Bio-Products, Woodland, CA, USA). Cultures were carried out for 20 or 72 h.

Bacterial inoculum resulting from overnight cultures was diluted in DMEM low glucose and grown aerobically at 37 °C to optical density 0.6 (~3 h). T cells were cultured in the absence or presence of bacteria at a multiplicity of infection of 100 for *E. coli* and 50 for *S*. Typhimurium [31]. For the proliferation assay, T cells labeled with carboxyfluorescein succinimidyl ester (CFSE) dye (10^–6^ M) were cultured in a 1:2 ratio for 5 days in serum-free AIM-V medium (Invitrogen, Carlsbad, CA, USA). The T cell proliferation induction by the bacteria was determined by evaluating the dilution of the fluorescence dye. The results express the percentage of the proliferating cells relative to maximum unspecific stimuli with OKT-3 (2.5 ng/mL) and rhIL-2 (150 iU/mL). T cells were seeded at 1 × 10^5^ cells per well (96-well format) in tissue culture plates coated with 5 mg/mL of anti-CD3 (clone 145-2C11; BioLegend, San Diego, CA, USA). T cells were cultured in the absence or presence of bacteria. After 2 h of incubation at 37 °C and 5% CO_2_, T cells were collected by centrifugation and the pellet was resuspended in medium supplemented with penicillin/streptomycin (2%) and gentamicin (50 mg/mL), killing all bacteria within 2 h. After an additional 18–20 h of incubation at 37 °C and 5% CO_2_, T cells were harvested, stained and analyzed by flow cytometry. Cells were stained with anti-CD3 APC (BD, Mountainview, CA, USA) for 20 min at 25 °C, washed in PBS with 0.01% BSA, fixed in 1% paraformaldehyde, then stored at 4 °C until analysis. Flow cytometric analysis was performed on a FACSVerse (Becton Dickinson) flow cytometer with Cell Quest Software (Becton Dickenson). Lymphocytes were identified by forward and side light scatter, and the frequencies of T cells with CFSE dye dilution were determined.

### 2.9. Statistical Analysis

In vitro adhesion was evaluated in three independent assays. Data from the adhesion assays were analyzed using Student’s t-test (two-tailed) or the Mann–Whitney test. T-lymphocyte proliferation was evaluated in three independent assays and the data were analyzed by one-way ANOVA. A P-value < 0.05 was considered statistically significant. All statistical analyses were performed in GraphPad Prism v. 7.00 (La Jolla, CA, USA).

## 3. Results

### 3.1. EF-Tu and l-Asparaginase II Are Secreted and Associated with the Surface of STEC O157:H7

**Figure 1:**
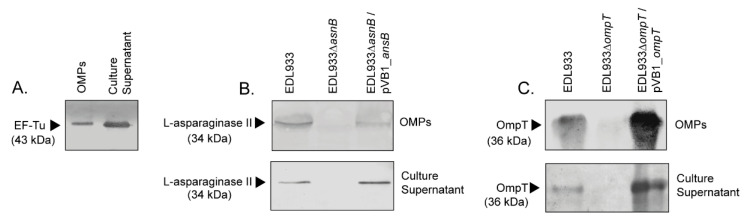
Determination of the presence of EF-Tu, l-asparaginase II and OmpT in outer membrane protein (OMP) extracts and culture supernatants. Protein extracts were separated by SDS-PAGE (12% acrylamide) and analyzed by Western blot assay. Arrows indicate the detection of the corresponding protein. (**A**) Identification of EF-Tu in OMPs and culture supernatant of the EDL933 strain. Monoclonal anti-EF-Tu antibody (mAb 900) was used in a dilution of 1:2000, followed by anti-mouse IgG, HRP conjugate diluted 1:5000. (**B**) Identification of l-asparaginase II in the OMP extracts and culture supernatant obtained from the EDL933, the isogenic mutant ELD933∆*ansB* and the complemented ELD933∆*ansB*/pVB1_*ansB* strains. Anti-l-asparaginase II antibody was used in a dilution of 1:2000, followed by anti-rabbit IgG, HRP conjugate diluted 1:5000. (**C**) Identification of OmpT in the OMP extracts and culture supernatant obtained from the EDL933, the isogenic mutant ELD933∆*ompT* and the complemented ELD933∆*ompT*/pVB1_*ompT* strains. Anti-OmpT antibody was used in a dilution of 1:3000, followed by anti-rabbit IgG, HRP conjugate diluted 1:5000.

**Figure 2:**
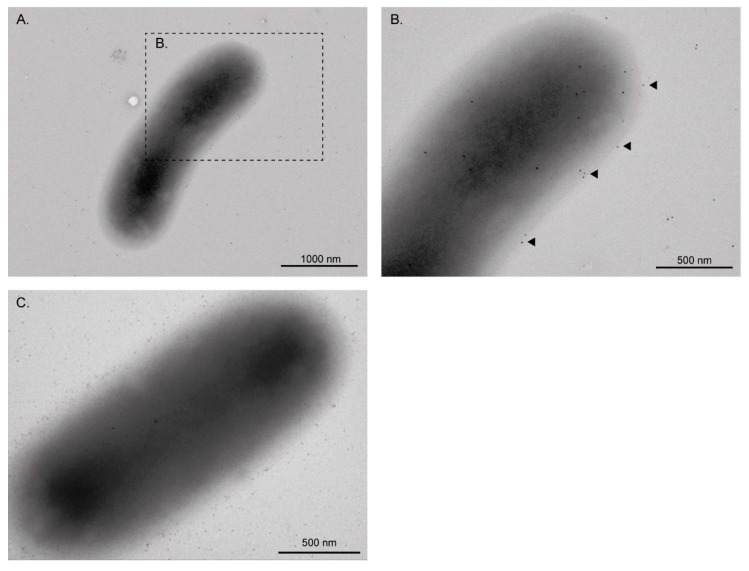
Immunogold electron microscopy showing detection of EF-Tu on the surface of STEC O157:H7 str. EDL933. (**A**,**B**) The localization of EF-Tu on the cell surface of EDL933 strain was demonstrated by abundant labeling with gold particles (Arrows). Bacteria were incubated with monoclonal anti-EF-Tu antibody (mAb 900; 1:200) followed by anti-mouse IgG+IgM (1:100) 10 nm gold particles. (**C**) As the control, the EDL933 strain was also incubated with anti-mouse IgG+IgM (1:100) gold particles. Note that in this case, a few gold labels were observed, but they appear to correspond to background noise rather than a specific detection.

In a previous study we identified EF-Tu in the OMP extract of the STEC O157:H7 str. E030-00, which is a clinical strain isolated from a HUS case [10]. Therefore, we asked whether EF-Tu is also present in the OMP extract of other strains of this serotype. For this, we analyzed the OMP extract of the STEC O157:H7 str. EDL933, which is a widely studied reference strain. As shown in Figure 1A, a Western blot analysis confirmed the presence of EF-Tu in the OMP extract and culture supernatant of the EDL933 strain. We next sought to confirm the localization of this protein on the bacterial surface using transmission electron microscopy (TEM). As expected, immunogold labeling of EF-Tu revealed individual or clustered gold particles on the surface of the EDL933 strain (Figure 2), indicating that EF-Tu is a surface-associated protein in this bacterium.

**Figure 3:**
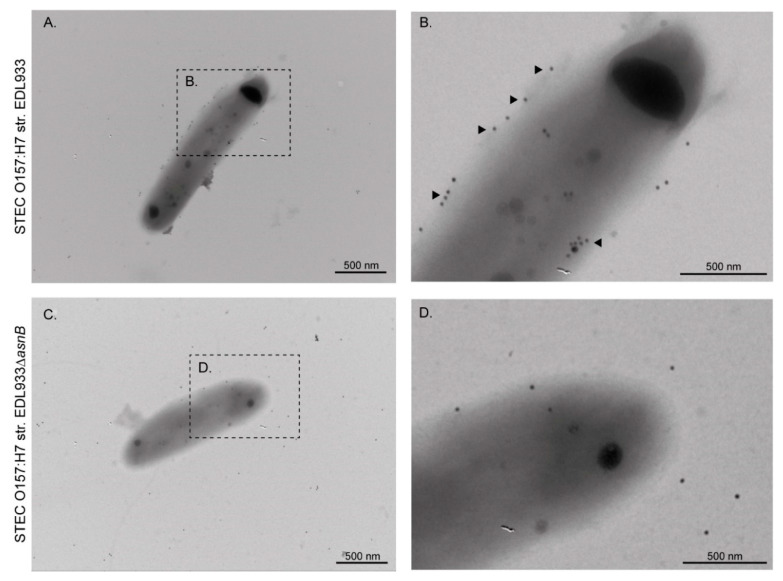
Immunogold electron microscopy showing detection of l-asparaginase II on the surface of STEC O157:H7 str. EDL933. Bacteria were incubated with anti-l-asparaginase II (1:200) followed by anti-rabbit IgG+IgM (1:100) 20 nm gold particles. (**A**,**B**) Localization of l-asparaginase II on the surface of the EDL933 strain. The localization of l-asparaginase II on the cell surface was demonstrated by abundant labeling with gold particles (Arrows). (**C**,**D**) Image obtained from the EDL933∆*asnB* strain. Note that in this case, a few gold labels were observed, but they appear to correspond to background noise rather than a specific detection.

Similarly, l-asparaginase II was identified in the OMP extract and culture supernatant of the EDL933 strain but not in its isogenic derivative ∆*ansB* strain (Figure 1B). When the ∆*ansB* strain was complemented with the plasmid pVB1_*ansB,* a reactive band was again observed in the OMP extract and culture supernatant. We also confirmed by immunogold labeling the presence of l-asparaginase II on the surface of the EDL933 strain, whereas the surface of the ∆*ansB* strain was virtually devoid of gold labeling (Figure 3).

The identification of EF-Tu and l-asparaginase II in the culture supernatant raises the question of how these proteins are secreted by STEC. A proteomic analysis of OMVs showed the presence of EF-Tu but not l-asparaginase II (Table 2). Thus, this result indicates that EF-Tu is part of the protein cargo released via OMVs. By contrast, the export mechanism by which l-asparaginase II is secreted remains to be identified.

### 3.2. The Monoclonal Anti-EF-Tu Antibody (mAb 900) Does Not Affect the Adhesion of STEC O157:H7 str. EDL933 to HT-29 Epithelial Cells, Fibronectin and Laminin

It has been reported that the EF-Tu of many bacterial species is involved in adhesion to human epithelial cells and to extracellular matrix proteins [15,16,17,18,19,20,21,22,23]. *E. coli* has two copies of the *tuf* gene (*tufA* and *tufB*), which are almost identical but located in different chromosomal regions, allowing the deletion of one copy without loss of cell viability [51]. However, because of the essential biological function of EF-Tu, deletion of the two gene copies is lethal to the bacterium. Therefore, to determine whether EF-Tu has a role in the adhesion of the EDL933 strain, we investigated whether the monoclonal anti-EF-Tu antibody 900 (mAb 900, HycultBiotech) affects the adherence of this bacterium to HT-29 cells, fibronectin and laminin. As shown in Table 3, the adherence levels of the EDL933 strain were unaffected when the bacterium was preincubated with the mAb 900 at dilutions of 1:10 and 1:100.

### 3.3. l-Asparaginase II Produced by STEC O157:H7 str. EDL933 Inhibits T Cell Proliferation

**Figure 4:**
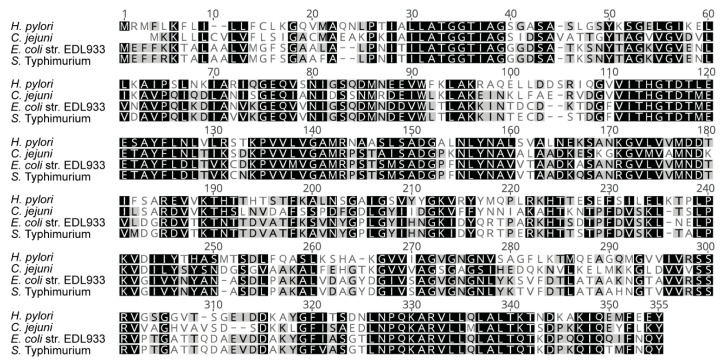
Amino acid sequence alignment of the l-asparaginase II homologues from different bacterial species. Alignment was performed using MUSCLE [54]. The accession numbers of the sequences used are AAG58088.1 (EDL933 strain), WP_128026528.1 (*Helicobacter pylori*), WP_084766606.1 (*Campylobacter jejuni*) and AAL21981.1 (*Salmonella* Typhimurium).

L-asparaginase II secreted by *S.* Typhimurium contributes to evasion of the host immune system in a mechanism that involves the deprivation of exogenous L-asparagine to limit T cell proliferation [31]. Similarly, l-asparaginase secreted by *Helicobacter pylori* functions as a cell-cycle inhibitor of fibroblasts and gastric cell lines [52], whereas periplasmic asparaginase of *Campylobacter jejuni* allows this bacterium to utilize asparagine and to colonize the liver more efficiently [53]. Therefore, as a first approach, we compared the amino acid sequence of the l-asparaginase II produced by the EDL933 strain and the homologues produced by the abovementioned pathogens. As a result, we found that the l-asparaginase II produced by the EDL933 strain has 51%, 56% and 93% amino acid identity with the homologues produced by *H. pylori, C. jejuni* and *S.* Typhimurium, respectively (Figure 4).

**Figure 5:**
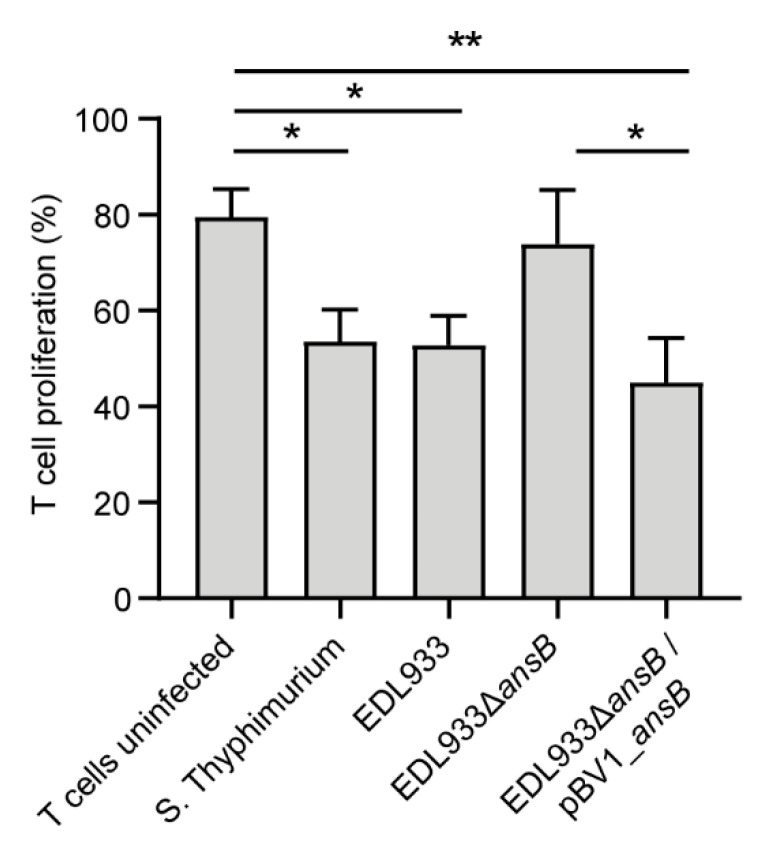
Proliferation of CFSE-labeled T cells. T cells were left uninfected (negative control) or cultured with the EDL933, EDL933∆*asnB* and EDL933∆*asnB*/pVB1_*ansB* strains. As a positive control, T cells were cultured with *S.* Typhimurium. Error bars show standard deviation (SD). Data were analyzed by one-way ANOVA. * *p* < 0.05, ** *p* < 0.005.

Due to the high amino acid identity with the l-asparaginase II produced by *S.* Typhimurium, we set out to determine whether the homologue produced by the EDL933 strain may also inhibit T cell proliferation. As shown in Figure 5, the CFSE-labeled T cells cultured with *S.* Typhimurium or the EDL933 strain did not proliferate in response to the stimulus. Conversely, uninfected CFSE-labeled T cells or those cultured with the EDL933∆*asnB* proliferated significantly. The complementation of the EDL933∆*asnB* strain with pVB1_*ansB* restored the capacity of the bacterium to inhibit T cell proliferation. This result indicates that, as reported for *S.* Typhimurium, l-asparaginase II produced by the EDL933 strain plays a role in evasion of the host immune system.

### 3.4. Deletion of The ompT Gene Affects The Adhesion of STEC O157:H7 str. EDL933 to HT-29 Cells

Recent studies have shown that the OmpT protein contributes to the adhesion of the ExPEC and APEC strains [39,40,41]. Consequently, it was interesting to evaluate whether OmpT also contributes to the adhesion of STEC. For this, we first confirmed that the EDL933 strain produces this protein. As a result, OmpT was identified by Western blot in OMP extracts and culture supernatant obtained from this strain (Figure 1C). As was observed for EF-Tu, OmpT was identified in the proteome of OMVs (Table 2), which may explain the identification of this protein in the supernatant. By contrast, no reactive protein band was observed in either the OMP extract or culture supernatant obtained from the isogenic-derivative ∆*ompT* strain. When the ∆*ompT* strain was complemented with the plasmid pVB1_*ompT,* a reactive band was again observed in the OMP extract and culture supernatant.

**Figure 6:**
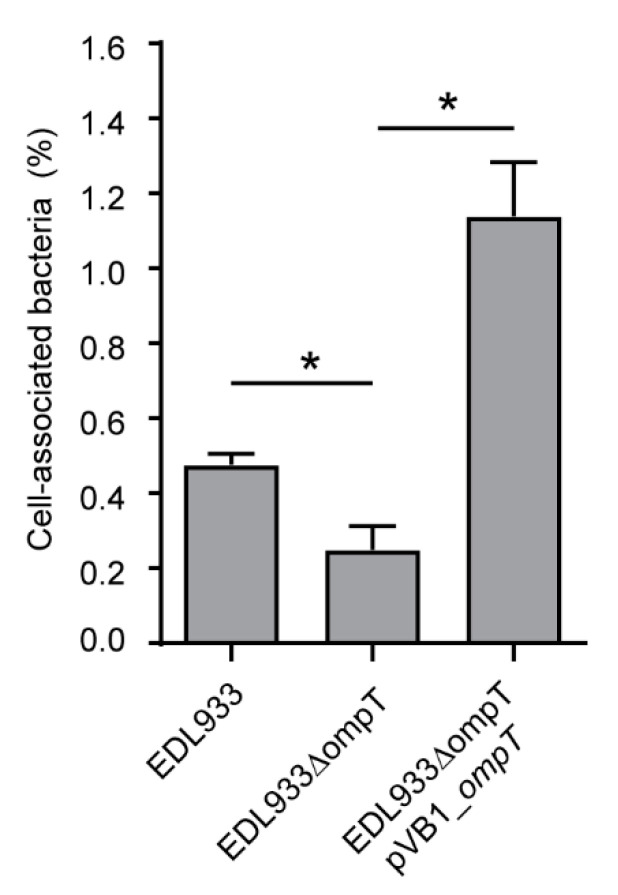
Participation of the OmpT protein in the adhesion of STEC O157:H7 str. EDL933 to HT-29 cells. Data are expressed as percentage of adhesion compared to total bacteria present after 3 h of infection at a multiplicity of infection (MOI) of 10 bacteria per cell. Error bars represent SD. (*n* = 3). * *p* < 0.05 by Student’s t-test (two-tailed) relative to the EDL933 strain.

Next, we sought to determine whether the deletion of the *ompT* gene affects the adhesion of the EDL933 strain to HT-29 cells. Notably, we found that the deletion of the *ompT* gene impairs the capacity of the EDL933 strain to adhere to HT-29 cells (Figure 6). Furthermore, the complementation of the EDL933∆*ompT* strain with pVB1_*ompT* restored the adhesion capacity and even enhanced it compared to the wild type strain.

## 4. Discussion

The first studies reporting the presence of EF-Tu in OMP extracts of *E. coli* were published in the 1970s and 1980s [55,56]. However, in this bacterium, the presence of EF-Tu in OMP extracts has been taken with caution and it has been largely attributed to contamination with cytoplasmic proteins [57,58,59]. Conversely, in other bacteria, a considerable amount of experimental evidence based on TEM and immunogold labeling has demonstrated the localization of EF-Tu on the cell surface [16,18,19,22]. In this context, a major finding of this study was to demonstrate the localization of EF-Tu on the surface of the EDL933 strain (Figure 2). Additionally, our comparative proteomic analysis showed the presence of EF-Tu in the OMVs of all three pathogenic *E. coli* studied here (Table 2). Thus, although as yet somewhat unclear, a possible export mechanism of EF-Tu could be through OMVs.

Different studies have shown the direct participation of EF-Tu in the adhesion of many bacterial species, supporting its classification as a moonlighting protein [5]. Therefore, a rational hypothesis is that this protein may have a similar role in STEC. However, an important limitation in testing this hypothesis is the impossibility of using traditional genetic manipulation methods, such as a knockout mutant of the two copies of the *tuf* gene [51]. Alternatively, we used a monoclonal anti-EF-Tu antibody in an attempt to block the possible adherence mediated by this protein. However, through this experimental approach we were unable to demonstrate any participation of EF-Tu in the adhesion of the EDL933 strain to HT-29 cells, fibronectin or laminin (Table 1). Further experiments using different anti-EF-Tu antibodies or alternative approaches could shed additional light on the role played by this protein on the surface of STEC.

By Western blot and immunogold labeling, we observed that l-asparaginase II is secreted, and that some molecules remain associated with the outer membrane of the EDL933 strain (Figure 3). However, this protein was not identified in the proteomic analysis of OMVs (Table 2). Moreover, this protein lacks a recognizable signal sequence for extracellular secretion and therefore the mechanism behind its transport to the surface is currently unknown.

The virulence roles reported to date for l-asparaginase proteins are a consequence of their enzymatic activities in asparagine catabolism, rather than an additional unrelated function [31,52,53,60]. Accordingly, we found that l-asparaginase II produced by the EDL933 strain contributes to pathogenicity by inhibiting T lymphocyte proliferation (Figure 5), most likely by mechanisms similar to those previously reported in *S*. Typhimurium [31]. Thus, this novel role for l-asparaginase II in the virulence of STEC is likely related to its known enzymatic activity.

Since EF-Tu and l-asparaginase II are located on the surface of the EDL933 strain, it is possible that during STEC infection these proteins could be recognized by the host immune system, triggering specific antibody responses. Consistent with this, a previous study showed that both proteins are reactive to convalescent sera from STEC-infected patients [10]. Overall, these findings suggest that STEC could use these proteins as accessory virulence factors during the infection in humans.

The proteolytic activity of OmpT is important for the virulence of STEC, participating in the degradation of antimicrobial peptides and biogenesis of OMVs [36,38]. Interestingly, our comparative proteomic analysis showed that OmpT is present in OMVs from STEC O157:H7 and AIEC NRG857c, but it is absent in OMVs from STEC O91:H21 (Table 2).

It has also been reported that this protein is also involved in adhesion in APEC and ExPEC strains [39,40,41]. However, the participation of OmpT in the adhesion of STEC had not been evaluated. As expected, our results showed that OmpT is also involved in the adhesion of STEC (Figure 6). Other proteases of the Omptin family are also involved in bacterial adhesion. For instance, the Pla protein has an important role in the adhesion of *Yersinia pestis* in a mechanism independent of its proteolytic activity [61]. Importantly, if the participation of OmpT in adherence is not related to its proteolytic activity, then it could be classified as a moonlighting protein. We will address this question in future studies. Thus, the OmpT protein participates in different mechanisms of pathogenicity in STEC and therefore is a promising target for vaccine development [62]. In fact, a recently developed vaccine candidate against STEC includes epitopes and antigenic domains of this protein [63]. Notably, this vaccine candidate confers protection on mice against the intestinal colonization and renal damage caused by STEC.

In conclusion, in the present study we identify possible additional (non-canonical) roles of the l-asparaginase II and OmpT proteins in the virulence of STEC. In the case of EF-Tu, we demonstrated its localization on the surface of STEC, but its role at this site is unknown. Our results extend the knowledge of the biological role played by these proteins in the pathogenic mechanisms of STEC.

## Figures and Tables

**Table 1:** Primers used in this study.

Primer Name	Sequence 5′ to 3′ *	Protocol	Source
d-ompT_F	ATCATTAAAACGATTGAATGGAGACCTTTTATGCGGGCGAAACTT CTGGGAATAGTCCTGACAACCCCTA**GTGTAGGCTGGAGCTGCTTC**	Deletion of the ompT gene	This work
d-ompT _R	CCAACCGGGGAGAAAATCTGGTTAACTTCGTTAAAAGGTGTACTT AAGACCAGCAGTAGTGATGAAGTTA**CATATGAATATCCTCCTTAG**	Deletion of the ompT gene	This work
ompT-e _F	TCCAGCATATTAAACCACAACAATAATA	Confirmation of the ompT deletion	This work
ompTc_F	CATATGCGGGCGAAACTTCT	Cloning of the ompT gene	This work
ompTc_R	GGATCCTTAAAAGGTGTACTTA	Cloning of the ompT gene	This work
d-ansB _F	AAGGGATAATGCGTAGCGTTCACGTAACTGGAGGAATGAAATGGA GTTTTTCAAAAAGACG**GTGTAGGCTGGAGCTGCTTC**	Deletion of the ansB gene	This work
d-ansB _R	TGAAGTGAAAAAGCCCCGGCGCGATACCGGGGCGAAATGATTAGT ACTGATTGAAGATCTG**CATATGAATATCCTCCTTAG**	Deletion of the ansB gene	This work
ansB-e _F	CCTCAATCCAAACCGAGTGGAAA	Confirmation of the ansB deletion	This work
ansBc_F	CATATGATGGAGTTTTTCAAAAAGACGGCAC	Cloning of the ompT gene	This work
ansBC_R	GGATCCTTAGTACTGATTGAAGATCTGCTGG	Cloning of the ompT gene	This work
kanR_R	GCCATGATGGATACTTTCT	Confirmation of allelic replacement	[50]

* The P1 and P2 priming sites are indicated in bold, according to Datsenko and Wanner [45]. Restriction sites are underlined.

**Table 2:** Identification of EF-Tu and OmpT proteins in outer membrane vesicles purified from Shiga toxin-producing *Escherichia coli* (STEC) O157:H7, STEC O91:H21 and AIEC NRG857c, by UPLC-MS/MS.

Protein Name	Description	Coverage Percentage	No. of Peptides	Protein Score *	Protein Intensity Values		
					STEC O157:H7	STEC O91:H21	AIEC NRG857c
A0A0H3EMH4	EF-Tu	69.04	27	1184.8	4.5 × 10^14^	1.6 × 10^15^	1.0 × 10^15^
A0A0H3EPM6	EF-Tu	69.04	27	1184.8	4.5 × 10^14^	1.6 × 10^15^	1.0 × 10^15^
A0A0H3ERC0	OmpT	14.20	6	124	1.6 × 10^13^	0	3.8 × 10^13^

* Protein Score (Mascot score), which is a probability-based implementation of the Mowse algorithm.

**Table 3:** Adherence of the EDL933 strain to HT-29 cells, fibronectin, and laminin in the presence of a monoclonal anti-EF-Tu antibody (mAb 900).

Experiment	HT-29 Cells		Fibronectin		Laminin	
	% Bacteria ± SD	*p* Value *	% Bacteria ± SD	*p* Value *	% Bacteria ± SD	*p* Value *
No antibody	100 ± 9.9	-	100 ± 7.3	-	100 ± 10.4	
Anti-EF-Tu (1:10)	107.7 ± 6.0	>0.05	118 ± 23.3	>0.05	135.2 ± 3.9	>0.05
Anti-EF-Tu (1:100)	105.4 ± 17.2	>0.05	Not evaluated	-	Not evaluated	-
Mouse IgG (1:10) **	113.6 ± 5.6	>0.05	131.9 ± 10.1	>0.05	133.8 ± 20.6	>0.05
Mouse IgG (1:100) **	121.1 ± 10.6	>0.05	Not evaluated	-	Not evaluated	-

* *p* value was determined by Mann–Whitney test, comparing the infection with no antibody to those containing different dilutions of antibodies. ** Purified mouse IgG was used as control.
